# Clonal Evolution Dynamics in Primary and Metastatic Lesions of Pancreatic Neuroendocrine Neoplasms

**DOI:** 10.3389/fmed.2021.620988

**Published:** 2021-05-05

**Authors:** Zhou Tong, Lin Wang, Weiwei Shi, Yanwu Zeng, Hangyu Zhang, Lulu Liu, Yi Zheng, Chunlei Chen, Weiliang Xia, Weijia Fang, Peng Zhao

**Affiliations:** ^1^Department of Medical Oncology, The First Affiliated Hospital, Zhejiang University School of Medicine, Hangzhou, China; ^2^Zhejiang Provincial Key Laboratory of Pancreatic Disease, The First Affiliated Hospital, Zhejiang University School of Medicine, Hangzhou, China; ^3^OrigiMed, Shanghai, China; ^4^State Key Laboratory for Diagnosis and Treatment of Infectious Disease, Collaborative Innovation Center for Diagnosis and Treatment of Infectious Diseases, The First Affiliated Hospital, Zhejiang University School of Medicine, Hangzhou, China; ^5^Division of Hepatobiliary and Pancreatic Surgery, Department of Surgery, The First Affiliated Hospital, Zhejiang University School of Medicine, Hangzhou, China

**Keywords:** clonal evolution, pancreatic neuroendocrine neoplasms, heterogeneity, liver metastasis, next-generation sequencing

## Abstract

**Background:** Data on inter-tumoral heterogeneity and clonal evolution of pancreatic neuroendocrine neoplasms (panNENs) with liver metastasis are limited. The aim of this study was to explore different patterns of clonal evolution of pancreatic neuroendocrine neoplasms with liver metastasis and the possible distinctive signaling pathways involved between G2 neuroendocrine tumors (NETs) and neuroendocrine carcinomas (NECs).

**Methods:** Tumor tissues of five patients (10 samples) with pancreatic neuroendocrine neoplasms with synchronous liver metastasis were analyzed using next-generation sequencing. PyClone, Gene Ontology, and Reactome pathway enrichment analysis were also applied.

**Results:** Mutated genes varied in individuals, reflecting the inter-tumoral heterogeneity of panNENs. The distribution of subclones varied during tumor metastasis, and different clonal evolution patterns were revealed between NETs and NECs. Gene Ontology and Reactome analyses revealed that in both NETs and NECs, signaling pathways and biological processes shared similarities and differences in the primary and metastatic lesions. In addition, the signaling pathway features were different between NETs and NECs. In the primary lesions, epigenetic changes and post-transcriptional modifications participated in NETs, while FGFR signaling, EGFR signaling, and NTRK2 signaling were largely involved in NECs. Although DNA repair and TP53 regulation were both involved in the metastatic lesions, most of the signaling pathways and biological processes disrupted by the mutated genes were different.

**Conclusions:** Our study revealed spatial inter-tumoral heterogeneity and temporal clonal evolution in PanNENs, providing potential therapeutic targets for further prospective clinical trials.

## Introduction

Pancreatic neuroendocrine neoplasms (panNENs) are heterogeneous tumors with distinct clinical syndromes and malignant potential. Unfortunately, the worldwide incidence and prevalence of panNENs have recently increased (1, 2). A subset of primary panNENs are aggressive and have a potential to metastasize. The 2019 World Health Organization (WHO) classification divides gastroenteropancreatic NENs into six groups—well-differentiated NETs G1, well-differentiated NETs G2, well-differentiated NETs G3, poorly-differentiated NECs—according to mitotic count and Ki-67 proliferation index, MiNEN and tumor-like lesions. Increased mitotic rate and high Ki-67 index are associated with a more aggressive clinical course and poorer prognosis (3, 4). Many studies have shown that distant metastases are related to poor overall survival in PanNENs (5, 6); however, the potential mechanisms of metastasis are poorly investigated and remain unclear.

Tumors progress and metastasize under Darwinian selection and evolution (7), and diverse genetic alterations create intra- and inter-tumoral heterogeneity. Difficulties with treatment, including drug resistance due to inter-tumoral heterogeneity and clonal evolution dynamics, are common. Some studies have shown concordance in mutational status between primary and distant metastases, where others do not (8, 9). Recent studies of breast cancer lung metastasis in mice showed that metastatic lesions were polyclonal, while omental metastases of high grade serous human ovarian cancer predominantly exhibited a single phylogenetic clade (10, 11). However, there are few studies investigating the clonal variation between primary and metastatic lesions of panNENs, or the different metastatic mechanisms among different grades. Studies of inter-tumoral heterogeneity in primary and metastatic lesions of panNENs are of great importance and could lead to more accurate clinical strategies for treatment.

The advent of next-generation sequencing (NGS) has increased understanding of tumor heterogeneity and clonal evolution (12, 13). In our study, we used a 450 oncogene sequencing panel to sequence pairs of primary tumors and hepatic metastases from synchronously metastasized panNENs from five patients. PyClone was applied to analyze the clonal populations and demonstrate the clonal distribution variation of primary and metastatic lesions. In addition, GO (Gene Ontology) and pathway enrichment analysis revealed distinct biological pathways that participated in primary and metastatic lesions in G2 neuroendocrine tumors (NETs) and neuroendocrine carcinomas (NECs).

## Materials and Methods

### Patients

Five panNEN patients with synchronous liver metastasis were included. Fresh tumor specimens were collected at the time of synchronous resection of primary tumors and liver metastases. Primary tissue samples, metastatic tissue samples, and adjacent normal tissue samples were collected. Our research was permitted by the Ethics Committee of the First Affiliated Hospital of Zhejiang University and the patients were informed of and gave consent to the use of tumor tissues for this research. All methods were performed in accordance with the declaration of Helsinki. Patient characteristics including patient demographics, pathologic TNM staging, histology stage, tumor thrombus, functional status, surgical approach, disease free survival (DFS), and patient status were obtained. TNM staging was adopted according to the 8th AJCC cancer staging system for neuroendocrine tumors of the pancreas. Grade was adopted according to the new WHO 2019 grading classifications.

### Hematoxylin and Eosin and Immunohistochemistry

Hematoxylin and eosin (HE) staining and immunohistochemistry against Ki67 were carried out on each sample to confirm diagnosis and to determine the histological subtype. Briefly, the tumor samples were fixed in 4% neutral paraformaldehyde, dehydrated via a gradient ethanol, and embedded within paraffin blocks. Then, the histological sections (5 um) were prepared, deparaffinized, hydrated, and subjected to HE staining and immunohistochemistry (IHC). For IHC, the hydrated sections were first heated in antigen retrieval buffer, permeabilized with PBS containing 0.1% Triton X-100, and then blocked with 1% bovine serum albumin (BSA). Then, the sections were incubated with a Ki67 primary antibody at 4°C overnight and treated with 3% H_2_O_2_ to inhibit endogenous peroxidases. Then, sections were incubated with an HRP-linked secondary antibody for 1 h at room temperature; 3,30-diaminobenzidine was used as a chromogenic agent. Finally, the nuclei were stained with hematoxylin before dehydration and mounting.

### Hybrid Selection and Sequencing

A custom hybridization capture panel including over 23,660 individually synthesized 5′-biotinylated DNA 120 bp oligonucleotides was used to target ~2.6 Mb of the human genome, including most exons of cancer-related genes and select introns of genes frequently rearranged in cancer (a 450 oncogene sequencing panel was used; gene names are listed in Supplementary Table 1). Hybridization capture employed xGen^®^ Lockdown^®^ Probes and Reagents (Integrated DNA Technologies, Version 3). Post-capture libraries were mixed, denatured, diluted, and then sequenced. For estimation of sequencing error rate, a PhiX spike-in was added as an external control to measure the percentage of reads with 0–4 mismatches, following the method described by Manley et al. (14). The average sequencing depth was 1000X for tissue-based deep sequencing.

### Bioinformatics Pipeline for SNV and Short Indels

Alignment of raw reads to the human genome reference sequence (hg19) was done with the Burrows-Wheeler Aligner (BWA, v0.6.2), followed by PCR duplicate removal using the MarkDuplicates algorithm from Picard (version 1.47, http://picard.sourceforge.net/). Local realignment and base quality recalibration for single nucleotide variants (SNV) were performed using GATK (v3.1-1) and subsequently culled by MUTECT (v1.7).

### Bioinformatics Pipeline for Copy Number Alternations

To identify CNA, aligned reads were first normalized within each bed by EXCAVATOR (version v2.2, http://sourceforge.net/projects/excavatortool/). Log ratio of read depths for each gene from tumor tissue and its matched normal blood control was then calculated. Tumor cellularity was estimated by allele frequencies of 4,088 sequenced SNPs (single nucleotide polymorphism), following the method in ASCAT.

### Bioinformatics Pipeline for Gene Rearrangement

For detection of gene rearrangement, aligned reads with abnormal insert size of over 2,000 or zero bp were collected and used as discordant reads, i.e., paired-end reads that could not be closely mapped to a genome reference, with each read of paired-reads aligned to the same chromosomes or different chromosomes. Originally, the discordant reads with the distance <500 bp formed clusters were further assembled by fermi-lite (https://github.com/lh3/fermi-lite) to identify potential rearrangement breakpoints. The breakpoints were double-confirmed by BLAT and the resulted chimeric gene candidates were annotated.

### Tumor Mutation Burden and Microsatellite Instability

Tumor mutation burden (TMB) was estimated by counting somatic mutations, including coding SNVs and indels per megabase of the sequence examined. Driver mutations and known germline alterations in dbSNP were not counted. MSI status was inferred based on MANTIS (15) score, and microsatellite regions were manually reviewed in Integrated Genomics Viewer (IGV) (16) for confirmation.

### PyClone

A Bayesian clustering method, PyClone, was used to infer clonal population structures present in the tumor as previously described (17). Briefly, given the mutation allele frequencies for each sample, PyClone estimates cellular prevalence for each cluster in each sample. PyClone is freely available for academic use at https://github.com/Roth-Lab/pyclone.

### GO and Reactome Pathway Enrichment Analysis

Related genes were selected using the Cytoscape GeneMANIA plugin (18). Genes from NEC and NET samples were combined separately and enrichment analysis for Biological Process was performed using the R software clusterProfiler (19) package with the pvalueCutoff set as 0.05. Reactome pathway enrichment analysis was performed using the ReactomePA package (20) with the *p*-value cutoff set as 0.05. Only the top 20 entries with a minimum adjusted *p*-value for primary and metastatic sites were included in the dotplot.

## Results

### Patient Demographics

**Figure 1:**
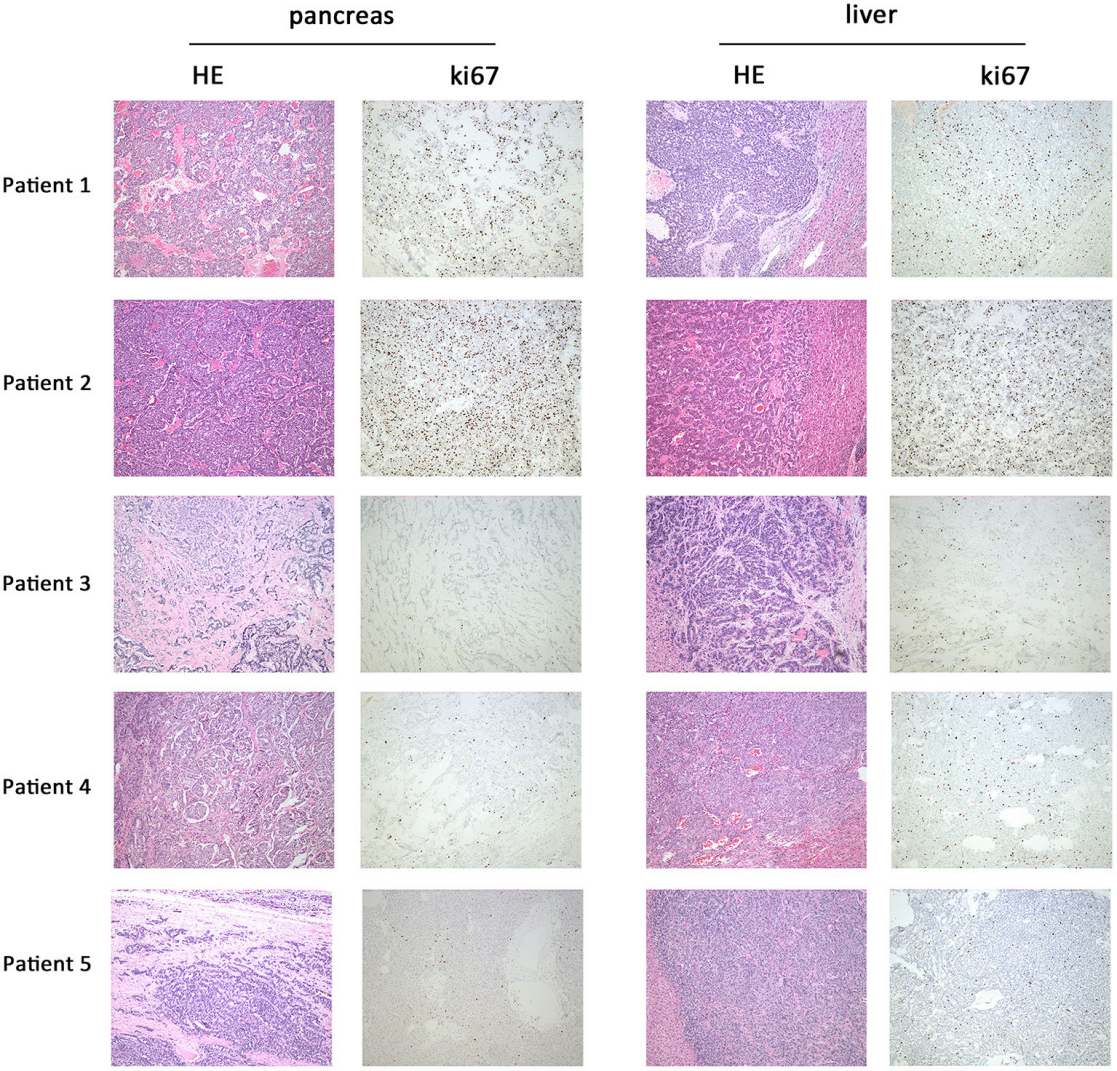
Representative hematoxylin and eosin and immunohistochemistry against ki67 in primary and metastatic lesions. In patient 1 and patient 2, HE staining of primary (pancreas) and metastatic lesions (liver) showed a mitotic count >20/10 HPF; the cells were poorly differentiated and composed of highly atypical neoplastic cells. Ki67 of patient 1 was 30–40%; patient 2 was 25–30%. In patients 3, 4, and 5, HE staining showed a mitotic count of 2–20/10 HPF. The cells were arranged in nests, glandular tubes, and were trabecular. Ki67 of patient 3 was 10–20%, patient 4 was 5–10%, and patient 5 was 8–10%. Magnification: 100x.

Five panNEN patients with synchronous liver metastasis were included. All patients received synchronous resection of primary and liver metastases. The pathology of patients 1 and 2 was NEC. In the primary and metastatic lesions, the cells were poorly differentiated, composed of highly atypical neoplastic cells, with a mitotic rate > 20/10 high-power fields, and ki67 > 20%. Patient 3, patient 4, and patient 5 were G2 NET. The cells were arranged in nests and glandular tubes, were trabecular, had a mitotic rate of 2–20/10 high-power fields, and ki67 3–20%. All tumors were positive for Synaptophysin and Chromogranin A. Representative hematoxylin- and eosin-stained sections and IHC against ki67 in primary tumors and metastases are shown in Figure 1. TNM stage is also listed. All tumors were non-functioning. The tumors of patients 1 and 2 exhibited perineural invasion, and patient 5 was confirmed to have tumor thrombus. Among the surgical choices for these patients, four patients were treated with distal pancreatectomy and one by pancreaticoduodenectomy. Liver tumor resection was performed in all cases. Mean follow-up time was 51.7 months (46.9–54.6 months). Patient characteristics are listed in Table 1.

**Table 1:** Characteristics of the five patients.

	**Age**	**Gender**	**Grade**	**Differentiated**	**T**	**N**	**M**	**Perineural invasion**	**Tumor thrombus**	**Surgical approaches**	**Function**	**DFS (mo)**	**Status**	**OS (mo)**
Patient 1	48	Male	G3	Poorly-differentiated	3	0	1	Yes	No	Pancreaticoduodenectoy + liver tumor resection	No	1.47	Deceased	51.8
Patient 2	61	Male	G3	Poorly-differentiated	3	1	1	Yes	No	Distal pancreatectomy + liver tumor resection	No	7.97	Deceased	45.9
Patient 3	70	Female	G2	Well-differentiated	4	0	1	No	No	Distal pancreatectomy + liver tumor resection	No	1.20	Alive	>53.8
Patient 4	59	Male	G2	Well-differentiated	2	0	1	No	No	Distal pancreatectomy + liver tumor resection	No	17.00	Alive	>50.1
Patient 5	71	Male	G2	Well-differentiated	3	1	1	No	Yes	Distal pancreatectomy + liver tumor resection	No	1.20	Alive	>53.4

### Sequencing

**Figure 2:**
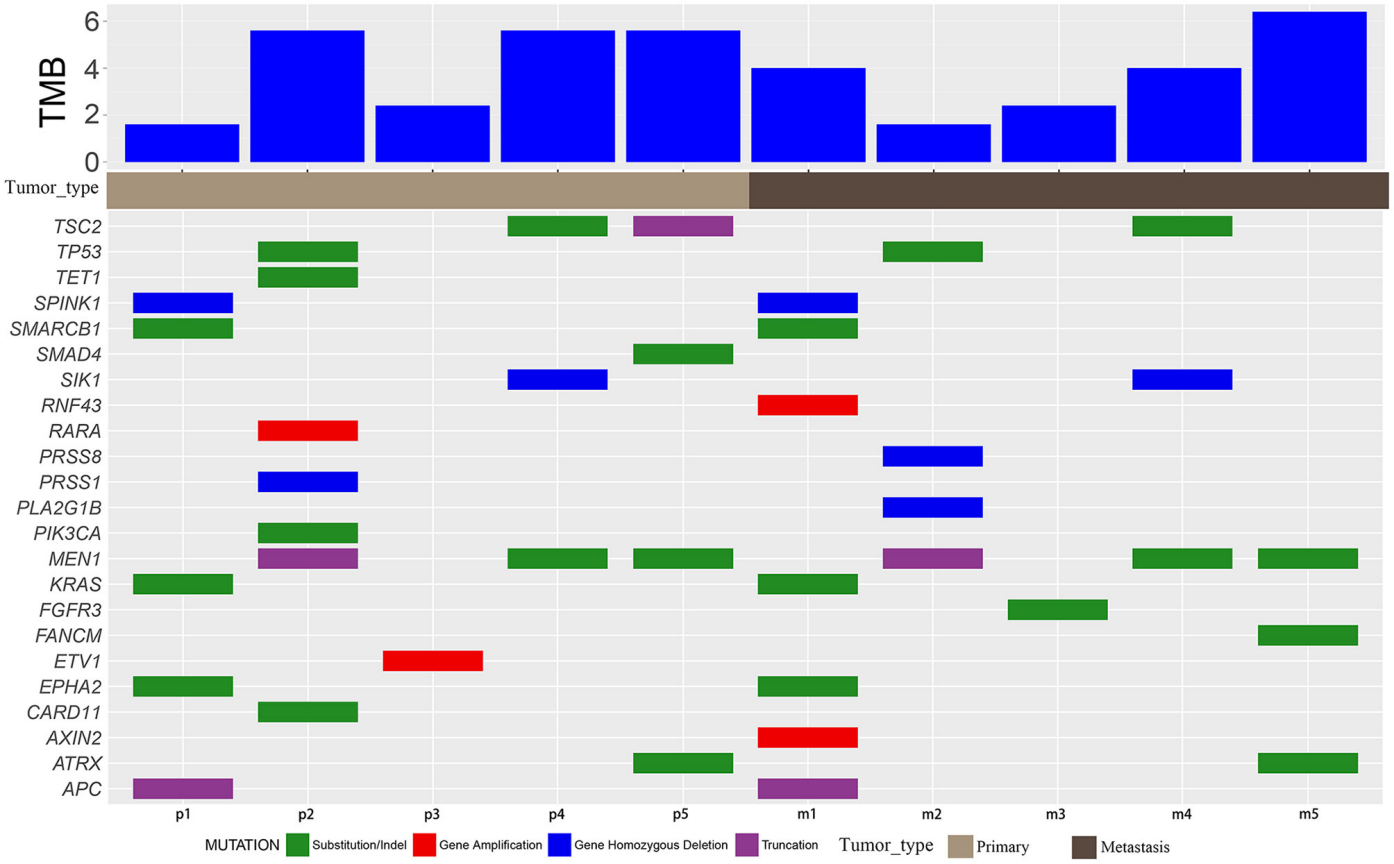
Mutation characteristics and TMB. Mutation details of primary (p) and metastatic (m) samples of patients 1–5. Green blocks represent substitution/indel. Red blocks represent gene amplification. Blue blocks represent homozygous gene deletion. Purple blocks represent truncation.

All primary and metastatic lesions of the five patients were sequenced (Figure 2, Supplementary Table 2) using a 450 oncogene sequencing panel. In patient 1, EPHA2, KRAS, SMARCB1, APC, and SPINK1 were altered in both primary and metastatic lesions, whereas RNF43 and AXIN2 were altered only in metastatic lesions. The VAF of EPHA2, KRAS, SMARCB1, APC all arose in metastatic lesion. In patient 2, TP53 and MEN1 were altered in both samples. Alteration of PIK3CA, RARA, CARD11, TET1, and PRSS1 were found only in the primary lesion while PRSS8 and PLA2G1B were found only in the metastatic lesion. The VAF of TP53 and MEN1 decreased in metastatic lesion. In patient 3, ETV1 was altered in the primary lesion and FGFR3 was altered in the metastatic lesion. In patient 4, MEN1, TSC2, and SIK1 were altered in both lesions and the VAF of TSC2 and MEN1 decreased in metastatic lesion. In patient 5, ATRX and MEN1 were altered in the primary and metastatic lesions. SMAD4 and TSC2 were altered in the primary lesion while FANCM was altered in the metastatic lesion. The VAF of ATRX arose from 0.53 to 0.57 in metastatic lesion while VAF of MEN1 decreased from 0.42 to 0.38. Mutated genes varied in the individuals, reflecting the heterogeneity of NETs and NECs. Compared with NETs, more mutations were revealed in NECs (more than five alterations in both primary lesions and metastatic lesions). And in NETs, gene alterations were less than four. After metastasis, the VAF elevated in one NEC patient, while the increasing VAF were hardly seen in NETs. In addition, in each patient, most of the mutated genes in the primary and metastatic lesions were common (except for patient 3). The TMB value varied from 1.6 to 6.4 mut/Mb in the five patients. All patients were classified as microsatellite stable (MSS).

### PyClone

**Figure 3:**
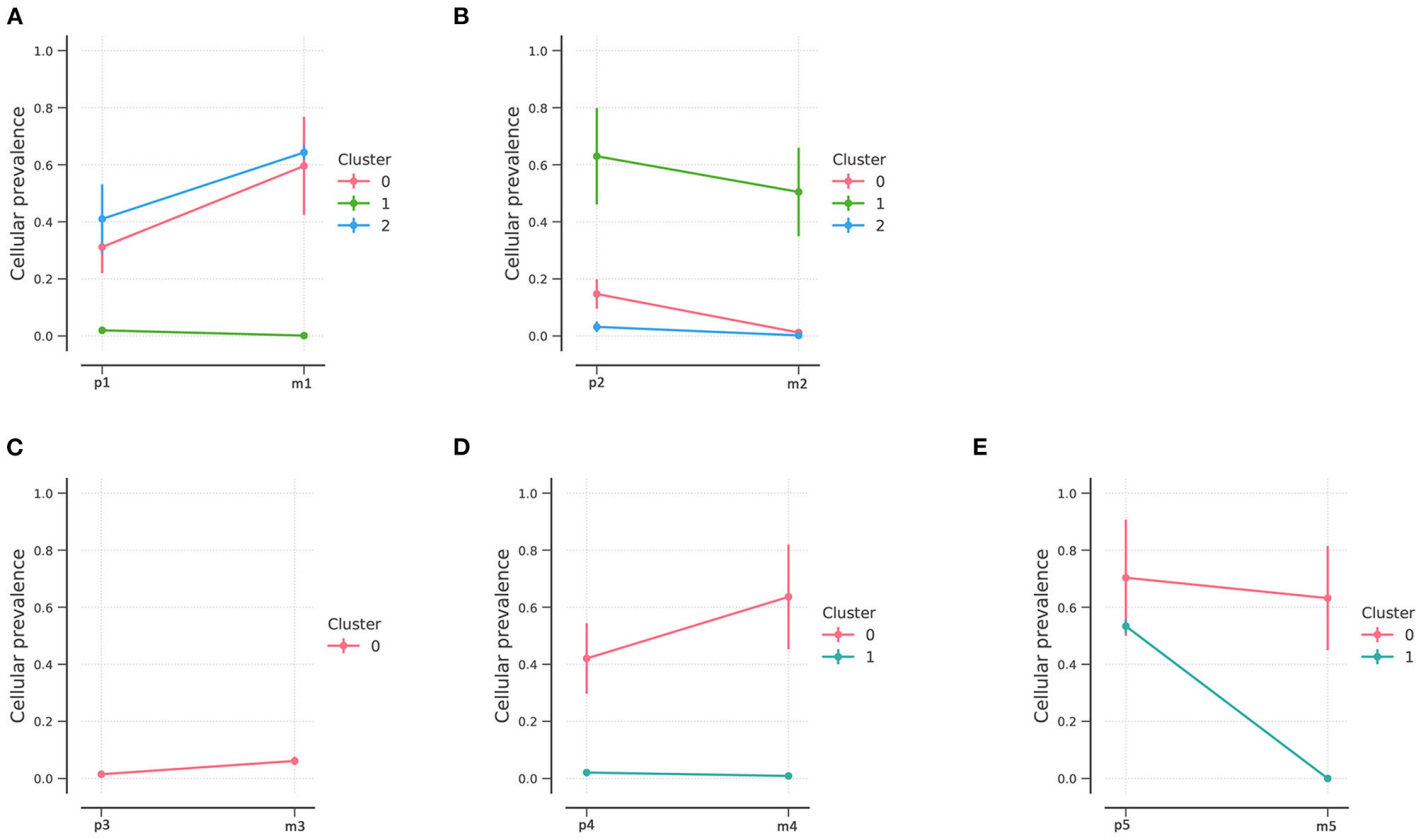
PyClone analysis of the five patients. PyClone quantification of clonal populations in sequenced mutations of patients 1–5 (**A–E**, respectively). Cellular prevalence of clonal populations is the mean value of the cellular prevalence of mutations in the cluster. The predicted cellular frequencies represent the proportion of cancer cells in each set of clonal mutation.

Based on variations identified using Bayesian clustering with PyClone, we identified three clusters in patients 1 and 2 (Figures 3A,B), one cluster in patient 3, and two clusters in patients 4 and 5 (Figures 3C–E); one cluster represents one subclone. Inter-tumoral heterogeneity was observed between the primary tumor samples and the metastases. The distribution of subclones changed, reflecting the evolution of tumors from primary to metastatic lesions. All tumor samples except for those of patient 3 showed evidence of subclonal structure.

NEC patients had three clonal populations in the primary lesions, while two clonal populations were identified in patient 1 and only one clonal population in patient 2. In addition, fewer clonal populations were found in G2 NETs and only one clonal population was identified in the metastatic lesions. Different clonal evolution patterns were identified between NETs and NECs.

### GO Term and Reactome Analysis

**Figure 4:**
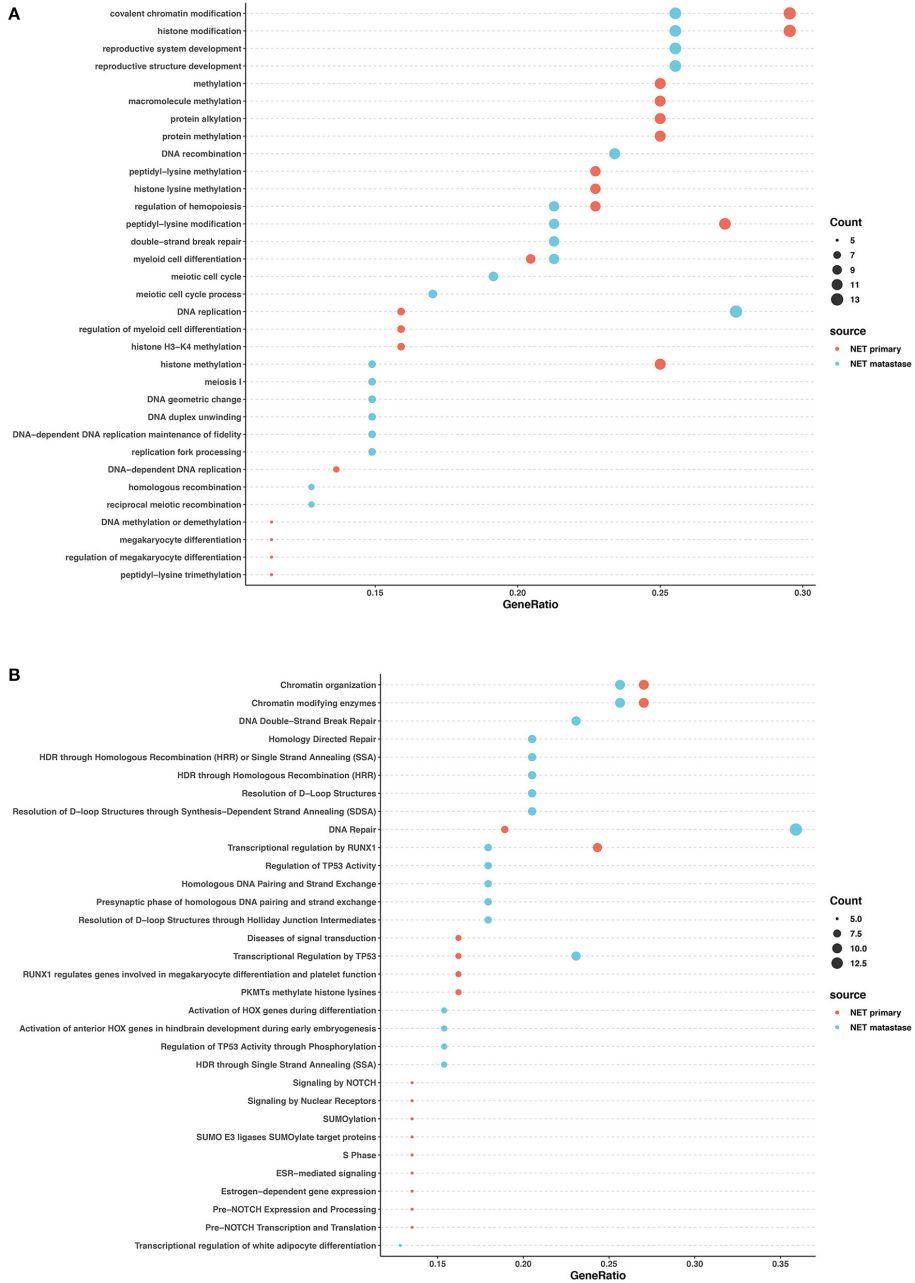
GO and pathway enrichment analysis of mutated genes involved in G2 NETs. GO enrichment analysis of biological process in mutated genes in NETs **(A)**. Reactome pathway enrichment analysis in NETs **(B)**. Dot size corresponds to number of genes; gene ratio is defined as percentage of genes in certain pathways compared with all genes in the samples.

We performed Gene Ontology (GO) enrichment analysis and Reactome analysis on sets of genes mutated in NETs and NECs. Mutated genes in the primary lesions of NET participated in sets of epigenetic changes and post-transcriptional modifications, such as histone methylation, chromatin modification, and macromolecule and protein methylation. In addition, the Reactome analysis showed that chromatin organization, SUMOylation, and the RUNX1 and NOTCH signaling pathways were most involved in the primary lesion. In the metastatic lesions of NETs, however, both Reactome and GO analysis showed that the mutated genes were prone to participation in histone and chromatin modification, DNA structure changes, DNA repair, and TP53 regulation. Histone and chromatin modification, DNA repair, and the regulation of TP53 and RUNX1 were involved in the primary and metastatic lesions in NET, whereas most other pathways were different (Figures 4A,B).

**Figure 5:**
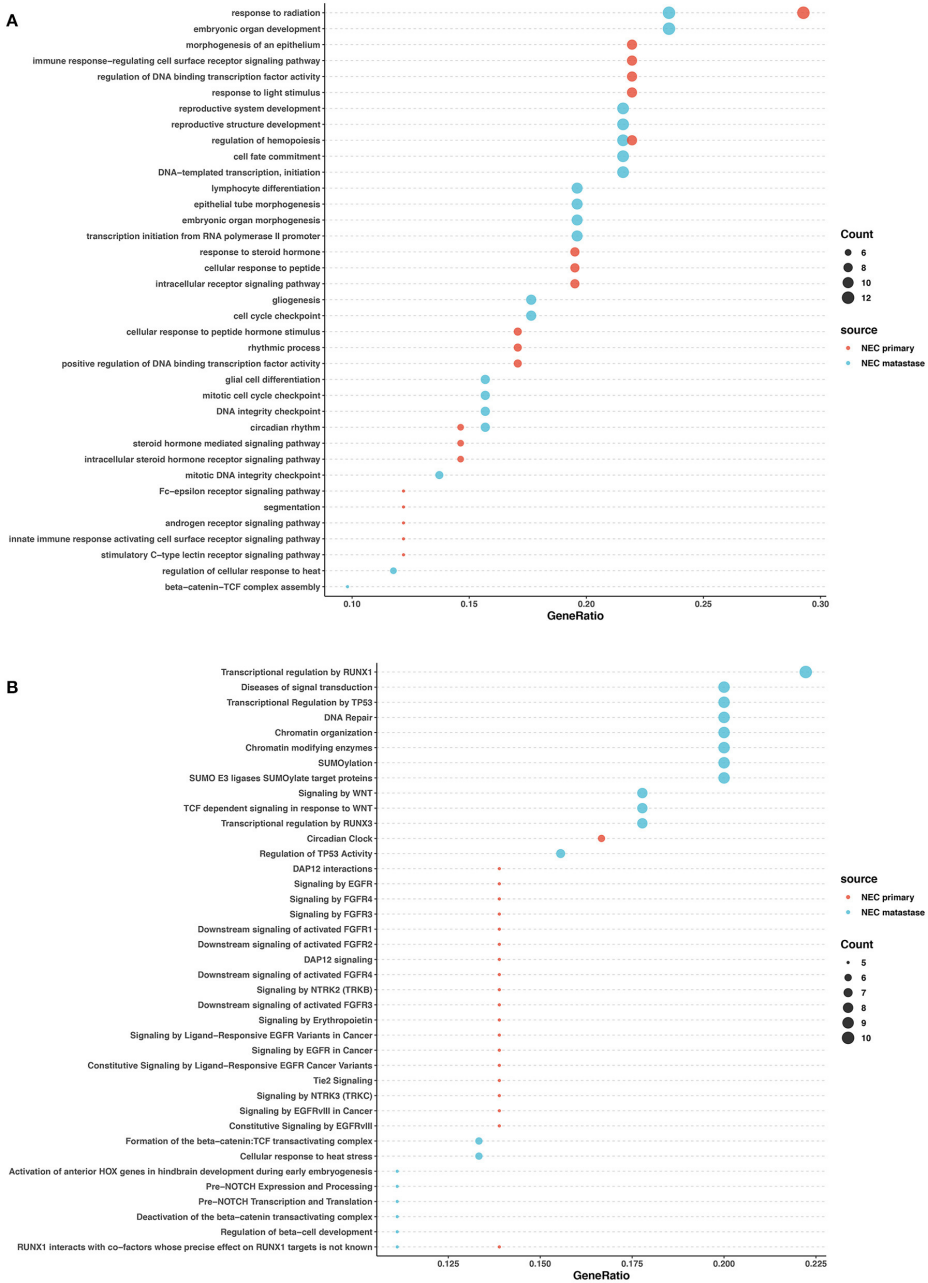
GO and pathway enrichment analysis of mutated genes involved in NECs. GO enrichment analysis of biological processes in mutated genes in NECs **(A)**. Reactome pathway enrichment analysis in NEC **(B)**. Dot size corresponds to number of genes; gene ratio is defined as the percentage of genes in certain pathways compared with all genes in the samples.

In the primary lesions of NECs, GO analysis revealed that the mutated genes were most related to radiation response, cellular response to peptides and hormones, and transcription factor activity regulation. The Reactome analysis found that FGFR signaling, EGFR signaling, and NTRK2 signaling were largely involved in the NEC primary lesions. In the metastatic lesions of NECs, GO analysis showed that the mutated genes largely participated in organ development and differentiation processes. Reactome analysis revealed that mutated genes influenced DNA repair, chromatin modification, SUMOylation, WNT signaling, and the regulation of transcription and activity of TP53 and RUNX1. The common pathways between the primary and metastatic lesions in NECs were rare except for radiation response (Figures 5A,B).

These data revealed that in both NETs and NECs, signaling pathways and biological processes shared similarities and differences in the primary and metastatic lesions. However, the signaling pathways and biological process patterns were different between NETs and NECs. In the primary lesion, epigenetic changes and post-transcriptional modification participated in NETs, while FGFR signaling, EGFR signaling, and NTRK2 signaling were largely involved in NECs. In the metastatic lesions, although DNA repair and TP53 regulation were both involved, most of the signaling pathways and biological process disrupted by the mutated genes were different between NETs and NECs.

## Discussion

Clonal evolution is defined by tumor heterogeneity over both space and time (21). Clonal evolutionary processes have been observed in many tumor types including pancreatic cancer (22), leukemia (23), and renal-cell carcinoma (24). Several large-scale genomic studies have characterized panNET genomes, including hundreds of somatic mutations and copy number variations, and reported that significantly mutated genes such as MEN1, DAXX, ATRX contribute to the mutagenic processes (25–27). Most panNECs harbor TP53 and RB1 alterations and lack neuroendocrine-related genetic changes (28). However, few researchers have studied the relationship between primary and metastatic lesions of individual panNENs from the perspective of clonal evolution. Our study performed NGS on five pairs of tumor lesions, revealing a comprehensive analysis of the course of tumor genomic evolution from primary lesion to metastatic lesion.

Tumors progress under Darwinian evolution, in which genetic variation alters molecular phenotypes in individual cells (29). Consequently, tumors often consist of multiple genes and different cell populations. These populations, known as clones, undergo selection in response to different tumor microenvironments or therapeutic interventions (30). Identifying dynamic clonal population structures can aid in predicting metastatic potential and chemotherapeutic resistance (17). PyClone is a Bayesian clustering method for grouping sets of somatic mutations into clonal clusters (17). PyClone was performed in the current study and identified three mutant clonal populations of primary lesions of panNEC and two clonal populations of panNET. Through the clone analysis, the sub-clones in each patient were identified, and the distribution of sub-clones reflected the evolutionary process of the tumors. Some clonal populations diminished, and some clonal populations expanded, which is one of the features of clonal evolution. Interestingly, metastatic lesions in NET patients tended to be from a single clone, which suggests monoclonal seeding from pancreatic lesion to liver and suggests a distinct clonal evolution mode from NEC. More importantly, the result should be confirmed by multiple lesion biopsies and NGS. It has been reported that panNETs exhibit a lower mutation burden (25), which may account for the small number of clonal populations in panNETs.

The failure of therapy and drug resistance is partially caused by intratumor heterogeneity, which provides diverse genetic material under evolutionary selection. In these five patients, the mutated genes varied in the individuals. The data from our study support the notion that pancreatic neuroendocrine carcinomas are fundamentally discordant from neuroendocrine tumors. Apart from their clinicopathological features, including histologic architecture, hormone production, and malignancy, the genetic profile differed. In patient 2, with the shortest overall survival, 45.9 months, TP53 was mutated in both the primary and metastatic lesions. It has been reported that in panNEC, ~70% of the tumors harbor TP53 mutations (28, 31, 32). Alternatively, in well-differentiated neuroendocrine neoplasms of the pancreas, TP53 mutations rarely occur. TP53 mutations, have been consistently associated with poor prognosis in cancers (33). Consistent with the literature, deleterious TP53 mutations were uncommon in panNET in our study. KRAS mutation was observed in one NEC patient and mutations in KRAS codon 12 were independently associated with a worse survival vs. wild-type KRAS (34). Somatic mutations of MEN1 occur in 30–44% of panNETs (25, 35, 36). In our study, patients 4 and 5 carried MEN1 mutations and patient 5 had a mutation of ATRX. DAXX and ATRX are mutually exclusive inactivating mutations, and no tumor with a mutation in ATRX had a mutation in DAXX. It has been reported that mutations in the MEN1 and the DAXX/ATRX genes are associated with prolonged survival compared with patients with tumors that lack these mutations (35). However, Fei Yuan et al. (37) and Marinoni et al. (38) reported that mutations of DAXX/ATRX were associated with a shortened survival. TSC2 mutations, which inhibit the mTOR signaling pathway and are mutually exclusive of mutations of PTEN, were revealed in patients 4 and 5. It has been reported that mTOR pathway genes (PTEN and TSC2) did not predict the pNET patients' survival (37). In addition, we observed high rates of common mutations in the primary and metastatc samples in these patients, except for patient 3. Recent studies on malignancies such as head and neck squamous cell carcinoma, non-small cell lung cancer, endometrial cancer, and small cell lung cancer all observed high rates of common mutations in primary and metastatic lesions (39–41).

Clonal evolution has two patterns, linear evolution or branched evolution. The linear model states that tumor cells acquire mutations over time, and that the strongest tumor becomes dominant. The branching evolution model states that parallel tumor cell clones acquire different genomic mutations over time (42, 43). In our study, both NEC and NET patients shared similar mutations in the primary and metastatic lesions, indicating the tumors were homogeneous at different locations and revealing a probable linear evolution model in PanNENs. This result should be confirmed by multiple biopsy analysis.

The mutated genes in the primary and metastatic lesions of NETs and NECs were analyzed via GO and Reactome analysis to explore the affected biological processes and pathways. The results revealed that the involved biological process and pathways were largely different among the primary and metastatic lesions of NETs and NECs. In NEC primary lesions, the EGFR, FGFR, and NTRK3 pathways were also involved. EGFR, FGFR, and NTRK3 targeted therapy may therefore be new options for NEC patients; however, clinical trials are required to validate this hypothesis. Further, mutated genes in the primary lesions of NETs exhibited sets of epigenetic changes, raising the intriguing possibility of epigenetic inhibitor treatment. Interestingly, though TP53 mutation was not detected in both primary and metastatic lesions in NETs, the Reactome analysis demonstrated that transcriptional regulation of TP53 was involved in NET primary lesions, whereas both the transcriptional regulation and activity regulation of TP53 were involved in NET metastasis. These results indicated that TP53 dysregulation may also play an important role in NET carcinogenesis and metastasis. In addition, we found that the DNA repair pathway participated in both NET and NEC metastasis. It has been reported that failed DNA repair can lead to carcinogenesis and tumor genome instability (44). And defects in DNA repair pathways may indicate a potential vulnerability to DNA-damaging therapies such as platinum (45). Additionally, oxaliplatin-based chemotherapy also shows a relatively high response rate in NETs (46). These findings not only provide better understanding of the mechanisms of NEC and NET carcinogenesis and metastasis, partially explaining the different clonal evolution patterns, but also give new insight into potential therapeutic approaches.

Immune checkpoint inhibitors have recently gained the attention of oncologists. PD-1/PD-L1 expression, tumor mutation burden, and DNA mismatch repair deficiency (dMMR) have been demonstrated as three potential biomarkers for the use of immune checkpoint inhibitors (47–50). Our results showed low TMB and MSS in both primary and metastatic lesions, which is consistent with other reports showing that neuroendocrine tumors have a relatively low mutation burden compared with other tumors (51) and may not benefit from immunotherapy.

This is the first study comparing primary and metastatic lesions of panNENs within the same person using NGS. Our study showed the mutation variation of primary and metastatic lesions and revealed clonal evolution dynamics in panNENs. We further detected different signaling pathways involved in the clonal evolution of NETs and NECs. However, our study had some limitations. The sample size was limited mostly due to the low incidence of PanNENs, and further studies including whole genome sequencing and other experimental studies are needed to verify the results. Our study does shed light on potential novel approaches for predicting and treating panNEN patients with synchronous liver metastasis.

## Conclusions

Our study revealed spatial inter-tumoral heterogeneity and temporal clonal evolution in PanNENs, which provides potential therapeutic targets for further prospective clinical trials.

## Data Availability Statement

According to national legislation/guidelines, specifically the Administrative Regulations of the People's Republic of China on Human Genetic Resources (http://www.gov.cn/zhengce/content/2019-06/10/content_5398829.htm, http://english.www.gov.cn/policies/latest_releases/2019/06/10/content_281476708945462.htm), no additional raw data is available at this time. Data of this project can be accessed after an approval application to the China National Genebank (CNGB, https://db.cngb.org/cnsa/). Please refer to https://db.cngb.org/, or email: CNGBdb@cngb.org for detailed application guidance. The accession code CNP0001685 should be included in the application.

## Ethics Statement

The studies involving human participants were reviewed and approved by Research Ethics Committee of the First Affiliated Hospital, College of Medicine, Zhejiang University (2017-778). The patients/participants provided their written informed consent to participate in this study.

## Author Contributions

ZT wrote the manuscript. LW, WS, and YZe analyzed the data. HZ and LL collected the clinical and pathological information from the cancer patients. YZh and CC designed the study. WF, WX, and PZ revised the manuscript. All authors contributed to the article and approved the submitted version.

## Conflict of Interest

WS and YZe were employed by the company OrigiMed. The remaining authors declare that the research was conducted in the absence of any commercial or financial relationships that could be construed as a potential conflict of interest. The reviewer H-KW declared a shared affiliation, though no other collaboration, with several of the authors, ZT, LW, HZ, LL, YZh, CC, WX, WF, and PZ to the handling editor.
