# Supplementary material for: Clonal Evolution Dynamics in Primary and Metastatic Lesions of Pancreatic Neuroendocrine Neoplasms
Source: Front Med (Lausanne). 2021 May 5;8:620988. doi: 10.3389/fmed.2021.620988 (PMC8131504; doi:10.3389/fmed.2021.620988)
Supplement: Supplementary file 1 [file Table_1.docx]

| **Supplementary Table 1. 450 Panel Current Gene List** | | | | | | | |
| --- | --- | --- | --- | --- | --- | --- | --- |
| **Target Therapy Genes** | | |  |  |  |  |  |
| ABL1 | ABL2 | ALK | ARAF | AXL | BCL2 | BRAF | BRCA1 |
| BRCA2 | BTK | CCND1 | CD274(*PD-L1*) | CDK4 | CDK6 | CDKN2A | CDKN2B |
| CSF1R | DDR1 | DDR2 | EGFR | EPHA2 | ERBB2(*HER2*) | ERBB3 | ERBB4 |
| FGFR1 | FGFR2 | FGFR3 | FGFR4 | FGR | FLT1 | FLT3 | FYN |
| HCK | HDAC9 | HGF | IGF1R | ITK | JAK1 | JAK2 | JAK3 |
| KDR | KIT | LCK | LIMK1 | LYN | MAP2K1(*MEK1*) | MAP2K2(*MEK2*) | MAP4K5 |
| MERTK | MET | MS4A1 | MST1R | MTOR | NEK11 | NTRK1 | NTRK2 |
| PDCD1(*PD-1*) | PDGFRA | PDGFRB | PIK3CA | PIK3CD | PTEN | PTK6 | RAF1 |
| RET | ROS1 | SIK1 | SMO | SRC | SRMS | TEK | TNFSF11 |
| TNFSF13B | TNK2 | TSC1 | TSC2 | VEGFA | YES1 |  |  |
|  |  |  |  |  |  |  |  |
| **Chemoradiotherapy Related Genes** | | | |  |  |  |  |
| APC | APEX1 | ARID1A | ARID1B | ATM | ATR | ATRX | BARD1 |
| BRCA1 | BRCA2 | BRIP1 | CHEK1 | CHEK2 | DAXX | EPCAM | ERCC1 |
| FANCA | FANCC | FANCD2 | FANCE | FANCF | FANCG | FANCL | FANCM |
| FEN1 | MGMT | MLH1 | MRE11A | MSH2 | MSH6 | NBN | PALB2 |
| PARP1 | PMS2 | POLB | PRKDC | RAD50 | RAD51 | RAD51B | RAD51C |
| RAD51D | RAD52 | RAD54B | RAD54L | RB1 | SMAD4 | TOP2A | WEE1 |
| XRCC2 | XRCC3 | ZNF217 |  |  |  |  |  |
|  |  |  |  |  |  |  |  |
| **Tumor Genetic Susceptibility Genes** | | | |  |  |  |  |
| APC | ATM | BARD1 | BLM | BMPR1A | BRCA1 | BRCA2 | BRIP1 |
| CDH1 | CFTR | CHEK2 | EPCAM | FANCA | FANCC | FANCD2 | FANCG |
| MEN1 | MLH1 | MRE11A | MSH2 | MSH6 | MUTYH | NBN | NF1 |
| NF2 | PALB2 | PMS2 | PRSS1 | PTEN | RAD50 | RAD51C | RAD51D |
| RB1 | RET | RHBDF2 | SDHA | SDHB | SDHC | SDHD | SMAD4 |
| SPINK1 | STK11 | TP53 | TSC1 | TSC2 | VHL | XRCC2 |  |
|  |  |  |  |  |  |  |  |
| **Rearrangement Genes** | | |  |  |  |  |  |
| ALK | AKT3 | BCL2 | BCR | BRAF | BRCA1 | BRCA2 | BRD4 |
| DDR2 | EGFR | ERBB2(*HER2*) | ERBB4 | ETV1 | ETV4 | ETV5 | ETV6 |
| EWSR1(*EWS*) | FGFR1 | FGFR2 | FGFR3 | JAK2 | KIT | MET | MSH2 |
| MYB | NOTCH1 | NOTCH2 | NRG1 | NTRK1 | NTRK2 | NTRK3 | PDGFB |
| PDGFRA | RAF1 | RARA | RET | ROS1 | SS18 | TMPRSS2 |  |
|  |  |  |  |  |  |  |  |
| **Tumor Related Genes (genes related to up/downstream in the target pathway, epigenetic regulation and prognosis prediction)** | | | | | | | |
| ACVR1B | ACVR2A | ADAM29 | ADGRA2 | AKT1 | AKT2 | AKT3 | AMER1 |
| AR | ARFRP1 | ARID2 | ASXL1 | ATF1 | AURKA | AURKB | AXIN1 |
| AXIN2 | BAP1 | BCL2L1 | BCL2L11(*BIM*) | BCL2L2 | BCL6 | BCOR | BCORL1 |
| BCR | BIRC5 | BLK | BMX | BRD4 | BTG1 | CAMTA1 | CARD11 |
| CBFB | CBL | CCND2 | CCND3 | CCNE1 | CD79A | CD79B | CDC73 |
| CDK12 | CDK8 | CDKN1A | CDKN1B | CDKN2C | CEBPA | CHD2 | CHD4 |
| CIC | COL1A1 | CRBN | CREB3L1 | CREB3L2 | CREBBP | CRKL | CRLF2 |
| CSF1 | CSK | CSNK1A1 | CTCF | CTNNA1 | CTNNB1 | CUL3 | CXCR4 |
| CYLD | CYP17A1 | CYP2D6 | DICER1 | DNMT3A | DOT1L | DPYD | EGF |
| EMSY | EP300 | EPHA3 | EPHA5 | EPHA7 | EPHB1 | ERG | ERRFI1 |
| ESR1(*ER*) | ETV1 | ETV4 | ETV5 | ETV6 | EWSR1(*EWS*) | EZH2 | FAM135B |
| FAM46C | FAS | FAT1 | FAT3 | FAT4 | FBXW7 | FEV | FGF10 |
| FGF12 | FGF14 | FGF19 | FGF23 | FGF3 | FGF4 | FGF6 | FGF7 |
| FH | FLCN | FLI1 | FLT4 | FOS | FOXL2 | FOXO1 | FOXP1 |
| FRS2 | FUBP1 | FUS | GABRA6 | GATA1 | GATA2 | GATA3 | GATA4 |
| GATA6 | GID4 | GLI1 | GLI2 | GLI3 | GNA11 | GNA13 | GNAQ |
| GNAS | GRIN2A | GRM3 | GSK3B | H3F3A | HNF1A | HRAS | HSD3B1 |
| HTATIP2 | HSP90AA1 | IDH1 | IDH2 | IGF2 | IKBKE | IKZF1 | IL7R |
| INHBA | INPP4B | IRF2 | IRF4 | IRS2 | JUN | KAT6A | KDM5A |
| KDM5B | KDM5C | KDM6A | KEAP1 | KEL | KLHL6 | KMT2A | KMT2C |
| KMT2D | KRAS | LMO1 | LRP1 | LRP1B | LRP2 | LZTR1 | MACC1 |
| MAGI2 | MAP2K4 | MAP3K1 | MAP3K13 | MCL1 | MDM2 | MDM4 | MED12 |
| MEF2B | MITF | MPL | MYB | MYC | MYCL | MYCN | MYD88 |
| NCOA2 | NCOR1 | NFE2L2 | NFIB | NFKBIA | NKX2-1 | NOTCH1 | NOTCH2 |
| NOTCH3 | NOTCH4 | NPM1 | NR4A3 | NRAS | NRG1 | NRG3 | NSD1 |
| NTRK3 | NUP93 | PAK3 | PARK2 | PARP2 | PARP3 | PARP4 | PAX5 |
| PBRM1 | PCA3 | PDCD1LG2 | PDGFB | PDK1 | PIK3C2B | PIK3CB | PIK3CG |
| PIK3R1 | PIK3R2 | PKD2 | PLA2G1B | PLCG2 | POLD1 | POLE | PPP2R1A |
| PRDM1 | PREX2 | PRKACA | PRKAR1A | PRKCI | PRSS8 | PTCH1 | PTK2 |
| PTPN11 | QKI | RAC1 | RANBP2 | RARA | RBM10 | RECQL | REL |
| RELA | RELB | RHOA | RICTOR | RNF43 | ROCK1 | ROCK2 | RPTOR |
| RUNX1 | RUNX1T1 | RXRA | SETD2 | SF3B1 | SLIT2 | SMAD2 | SMAD3 |
| SMARCA4 | SMARCB1 | SMARCD1 | SNCAIP | SOCS1 | SOX10 | SOX2 | SOX9 |
| SPEN | SPOP | SPTA1 | SS18 | SSX1 | STAG2 | STAT3 | STAT4 |
| STK24 | SUFU | SYK | TAF1 | TBX3 | TCF7L2 | TERT | TET1 |
| TET2 | TET3 | TFE3 | TGFBR1 | TGFBR2 | TIE1 | TIPARP | TMPRSS2 |
| TNFAIP3 | TNFRSF14 | TOP1 | TPMT | TSHR | TYK2 | U2AF1 | UGT1A1 |
| WEE2 | WHSC1 | WISP3 | WT1 | XIAP | XPO1 | ZBTB2 | ZNF703 |
| ZNF750 |  |  |  |  |  |  |  |
