# Supplementary material for: Clonal Evolution Dynamics in Primary and Metastatic Lesions of Pancreatic Neuroendocrine Neoplasms
Source: Front Med (Lausanne). 2021 May 5;8:620988. doi: 10.3389/fmed.2021.620988 (PMC8131504; doi:10.3389/fmed.2021.620988)
Supplement: Supplementary file 2 [file Table_2.docx]

| **ID** | **Gene** | **Transcript_Ref** | **Coding DNA change** | **aa change** | **VAF** | **COVERAGE** |
| --- | --- | --- | --- | --- | --- | --- |
| **p1** | *EPHA2* | NM_004431 | c.154-5delC |  | 0.176 | 420 |
|  | *KRAS* | NM_004985 | c.35G>A | p.G12D | 0.218 | 1013 |
|  | *SMARCB1* | NM_003073 | c.1097G>C | p.R366P | 0.194 | 811 |
|  | *APC* | NM_000038 | c.3340C>T | p.R1114* | 0.246 | 892 |
|  | *SPINK1* | NM_003122 | - | - | - | - |
| **m1** | *EPHA2* | NM_004431 | c.154-5delC |  | 0.344 | 320 |
|  | *KRAS* | NM_004985 | c.35G>A | p.G12D | 0.365 | 852 |
|  | *SMARCB1* | NM_003073 | c.1097G>C | p.R366P | 0.366 | 618 |
|  | *APC* | NM_000038 | c.3340C>T | p.R1114* | 0.643 | 561 |
|  | *RNF43* | NM_017763 | - | - | - | - |
|  | *AXIN2* | NM_004655 | - | - | - | - |
|  | *SPINK1* | NM_003122 | - | - | - | - |
| **p2** | *TP53* | NM_000546 | c.830G>T | p.C277F | 0.55 | 371 |
|  | *MEN1* | NM_130799 | c.252_253insGT | p.I85Vfs*35 | 0.36 | 1026 |
|  | *PIK3CA* | NM_006218 | c.3131A>G | p.N1044S | 0.02 | 547 |
|  | *MEN1* | NM_130799 | c.251_252insGG | p.I85Vfs*35 | 0.01 | 668 |
|  | *RARA* | NM_000964 | - | - | - | - |
|  | *CARD11* | NM_032415 | c.3144+7C>T |  | 0.11 | 394 |
|  | *TET1* | NM_030625 | c.4367+12C>A |  | 0.11 | 547 |
|  | *PRSS1* | NM_002769 | - | - | - | - |
| **m2** | *TP53* | NM_000546 | c.830G>T | p.C277F | 0.30 | 472 |
|  | *MEN1* | NM_130799 | c.252_253insGT | p.I85Vfs*35 | 0.20 | 890 |
|  | *MEN1* | NM_130799 | c.251_252insGG | p.I85Vfs*35 | 0.01 | 718 |
|  | *PRSS8* | NM_002773 | - | - | - | - |
|  | *PLA2G1B* | NM_000928 | - | - | - | - |
| **p3** | *ETV1* | NM_004956 | - | - | - | - |
| **m3** | *FGFR3* | NM_001163213 | c.1081+6G>A | - | 0.11 | 727 |
| **p4** | *TSC2* | NM_000548 | c.2640-1G>C |  | 0.37 | 704 |
|  | *SIK1* | NM_173354 | - | - | - | - |
|  | *MEN1* | NM_130799 | c.53_55del | p.D18del | 0.39 | 1335 |
| **m4** | *TSC2* | NM_000548 | c.2640-1G>C |  | 0.25 | 656 |
|  | *SIK1* | NM_173354 | - | - | - | - |
|  | *MEN1* | NM_130799 | c.53_55del | p.D18del | 0.25 | 1167 |
| **p5** | *ATRX* | NM_000489 | c.6769C>G | p.L2257V | 0.53 | 398 |
|  | *MEN1* | NM_130799 | c.784-3T>G | - | 0.42 | 576 |
|  | *SMAD4* | NM_005359 | c.788-2A>G | - | 0.27 | 559 |
|  | *TSC2* | NM_000548 | c.5068dup | p.D1690Gfs*16 | 0.05 | 1611 |
| **m5** | *ATRX* | NM_000489 | c.3809+3A>G | - | 0.57 | 457 |
|  | *MEN1* | NM_130799 | c.784-3T>G | - | 0.38 | 727 |
|  | *FANCM* | NM_020937 | c.6077T>C | p.I2026T | 0.06 | 643 |

Supplementary Table 2. Genomic alterations of primary and metastatic lesions of five patients.
